# Evaluation and Prediction of the Ecological Footprint and Ecological Carrying Capacity for Yangtze River Urban Agglomeration Based on the Grey Model

**DOI:** 10.3390/ijerph15112543

**Published:** 2018-11-13

**Authors:** Benhong Peng, Yuanyuan Wang, Ehsan Elahi, Guo Wei

**Affiliations:** 1 School of Management Science and Engineering, Nanjing University of Information Science & Technology, Nanjing 210044, China; 2 Research Institute of Climatic and Environmental Governance, Nanjing University of Information Science & Technology, Nanjing 210044, China; 3 School of Business, Nanjing University of Information Science and Technology, Nanjing 210044, China; ehsanelahi@nuist.edu.cn; 4 Department of Mathematics and Computer Science, University of North Carolina at Pembroke, Pembroke, NC 28372, USA; guo.wei@uncp.edu

**Keywords:** ecological footprint, ecological carrying capacity, grey model, Yangtze River urban agglomeration

## Abstract

The conflict between economic development and environmental protection has become increasingly prominent in the urbanization process of the Yangtze River urban agglomeration, the most economically developed region in Jiangsu Province in China. In order to investigate the sustainable development status, and thus provide decision support for the sustainable development of this region, the ecological footprint model was utilized to evaluate and analyze the ecological footprint per capita, the ecological carrying capacity per capita, and the ecological deficit per capita for the period from 2013 to 2017. Furthermore, the Grey model is employed to predict the development trend of the ecological footprint for 2018 to 2022. The evaluation results show that the ecological footprint per capita has been increasing year by year since 2013, reaching a peak of 2.3897 hm^2^ in 2015 before declining again. In the same period, the available ecological carrying capacity per capita and the ecological footprint per capita basically developed in the same direction, resulting in an ecological deficit per capita and gradually increasing from 2013 to a peak of 2.0303 hm^2^ in 2015 before declining. It is also found that the change of ecological carrying capacity is not substantial, and the change of the ecological deficit is mainly caused by a huge change of the ecological footprint. The forecast results show that the ecological deficit per capita will reach 1.1713 hm^2^ in 2018, which will be another deficit peak after 2015. However, in the later period until 2022, the ecological deficit per capita will begin to decline year by year. These results can provide effective inspirations for reducing the ecological deficit of the Yangtze River urban agglomeration, thus promoting the coordinated development of the economy and environment in this area.

## 1. Introduction

The conflict between the slow formation rate of natural resources and the growing demand for human beings is the core issue of regional and global sustainable development. According to the Earth Vitality Report 2014 [1], human beings need at least 1.5 times the amount of Earth’s resource regeneration capacity to provide for the total global consumption of ecosystem services, such as water pollution, and desertification, implying that human society faces severe, long-lasting challenges. For China, the level of urbanization has reached 57.3% in 2016 [2]. Urbanization has propelled the growth of national economies and is certain to be accompanied by unprecedented consumption and loss of natural resources [3]. Therefore, how to utilize the growth opportunities brought by urbanization and to facilitate China’s urban resources and the environment for sustainable development has become the focus of attention domestically and abroad.

The ecological footprint (*EF*) model is an effective way to measure sustainability. It was first proposed by the Canadian scholar Mathis Wackernagel in 1992 as a new theory and method to quantitatively measure the state of sustainable development based on the continuous dependence of human society on land [4].

In terms of the *EF* and ecological carrying capacity (*EC*), Wackemagel took the lead in applying the *EF* as early as 1997 to make national-level predictions about the human available ecological space and the already occupied ecological space [5,6]. Since then, some scholars have studied the regional *EF* at the regional scale [7,8,9,10,11,12,13]. Later, the *EF* was applied to tourism [14,15], energy [16,17,18,19], and other fields. Examples include incorporating the *EC* within the economic cost and benefit estimation to analyze crop production systems [20], combining those factors with the environmental Kuznets (EKC) hypothesis to study the relationship between environmental degradation and economic growth [21], and then adding the complexity assessment method of water ecosystem to study the reasons for the differences in the national water *EC* [22,23]. Some scholars also analyzed the regional resource and environmental carrying capacity based on the improved *EF* model [24,25,26].

In terms of the evaluation index system of the *EC*, domestic and foreign scholars mainly focus on the evaluation index system of the water ecological carrying capacity (W*EC*C) [27] and the evaluation index system of the marine *EC* [28] related to the *EF* of the water resources. At the same time, there are many methods used to evaluate the *EC* evaluation index system domestically and abroad. Most scholars in China adopted the analytic hierarchy process [29,30]. The advantage of this method is that it not only determines whether the current *EC* of the region is in a deficit, but also possibly derives ecological flexibility and investigates whether the environment is in a low- or high-pressure state.

To have a better understanding of the study area’s future *EC* and to provide a decision-making basis for sustainable development, scholars began to establish an evaluation and prediction model of the *EC* [31] and combined the *EF* method with the Autoregressive Integrated Moving Average (ARIMA) model [32] or the Grey model [33] to forecast the regional future *EC*s, and also combined it with the Stochastic Impacts by Regression on Population, Affluence, and Technology (STIRPAT) model [34,35,36] to study the drivers of *EF* changes.

However, the current evaluations and predictions of the *EF* and the *EC* are mostly concentrated at the national and regional levels, and there are only a few studies on the sustainability of urban scales and urban agglomerations scales. In addition, most of the articles that focus on static research only describe the current status of the *EF* or *EC* and pay less attention to the dynamic changes of the *EC*, which has resulted in less research on its prediction and makes it difficult to play a role in regional development decision-making. 

As an emerging national strategy, the evaluation and prediction of the *EC* of the Yangtze river urban agglomeration is conducive to better understanding the current status and development trend of the regional carrying capacity, and to realizing the sustainable development of its resources and environment, which is of positive significance for advancing China’s urbanization construction and the Belt and Road construction.

Therefore, we use the *EF* model to obtain the *EF* per capita and *EC* per capita of the Yangtze River urban agglomeration from 2013 to 2017 and to analyze the trend of the *EF*. On this basis, we develop a (1, 1) Grey model (GM) to predict the *EF* and *EC* for the next five years (2018–2022), to quantitatively judge the sustainable development, and to provide a decision-making basis for future sustainable development.

The rest of this paper is structured as follows. In Section 2, we introduce the research area, *EF* model, Grey model, and data sources. In Section 3, we investigate the *EF* and *EC* of the Yangtze River urban agglomeration from 2013–2017, and predict its *EF* and *EC* for 2018–2022. Finally, Section 4 concludes the study and raises some suggestions.

## 2. Methodology

### 2.1. Study Area

**Figure 1:**
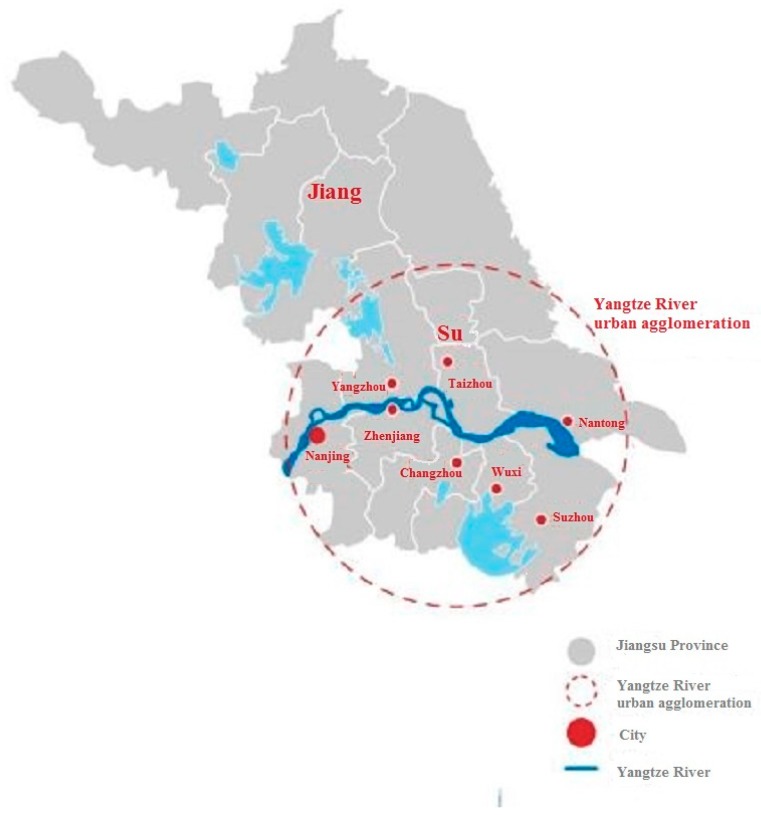
Map of Yangtze river urban agglomeration.

Yangtze River urban agglomeration is located across the national “Belt and Road” construction and the integration development of the Yangtze River Economic Belt, covering Nanjing, Suzhou, Wuxi, Changzhou, Zhenjiang, Nantong, Yangzhou, and Taizhou (Figure 1), accounting for almost half of the land area of the province, and creating approximately 80% of the total economy in the province. The agglomeration is the focus of economic development in Jiangsu province and is also the main position of economic belt construction in Yangtze River [37].

However, in recent years, with the acceleration of urbanization, the intensity of development, and utilization of various resources such as land has continued to increase, and the contradiction between economic development and environmental protection has gradually become prominent. Therefore, whether the ecological carrying capacity of the region can support its rapid development and population growth has become a national concern.

### 2.2. Ecological Footprint Model

#### 2.2.1. Calculation of the Ecological Footprint

We utilize the *EF* analysis method that was proposed by ecological economist Rees [7]. The *EF* is the sum of all kinds of land consumed by human activities, including six types of land: arable land, pasture, forest, built-up area, water area, and fossil-energy area. Using the equivalence factor, these six types of land are transformed into corresponding biologically productive areas that measure the pressure of the regional natural capital. The specific calculation formula is as follows:

(1) $$ef=\sum_{j=1}^{i}w_{j}\times A_{i}=\sum_{j=1}^{i}(w_{j}\sum \frac{c_{j}}{p_{j}}\times y_{j})$$

(2) EF=N×ef

where *ef* is the *EF* per capita; *j* is the type of productivity land; *i* is the category of the consumption item; *w_j_* is the equivalence factor; *Y_j_* is the yield factor; *A_i_* is the area of the consumption item; *c_j_* is the amount of consumption per capita of *i* item; *p_j_* is the local unit area yield of consumption item *I*; and *EF* and *N* are the total *EF* and population of a region, respectively.

#### 2.2.2. Calculation of the Ecological Carrying Capacity

The calculation formula of the *EC* is as follows [7]:

(3) $$EC=\sum_{j=1}^{n}w_{j}\times y_{i}\times A_{i}$$

(4) ec=EC/N

where *EC* is the total ecological supply, *ec* is the ecological supply per capita, and *y_i_* is the yield factor. It should be noted that the calculation result also needs to be deducted by 12% as land for biodiversity conservation.

#### 2.2.3. Calculation of the Ecological Surplus and the Ecological Deficit

The ecological surplus and the ecological deficit (ED) are used to reflect the utilization of natural resources by the population of the study area. When the *EF* exceeds the *EC*, an ED will be generated. In contrast, when the *EC* exceeds the *EF*, there will be an ecological surplus.

### 2.3. Grey Model

Based on the application of the *EF* model, we will establish the GM (1,1) grey model to predict the *EF* per capita, the *EC* per capita, and the ED per capita of Yangtze river urban agglomeration from 2018 to 2022, and quantitatively judge the sustainable development status of this region as a whole.

The Grey model prediction formula [38] is as follows:

(5) $${\hat{x}}^{\left(1\right)}\left(t+1\right)=\left(x_{\left(1\right)}^{\left(0\right)}-\frac{u}{a}e^{-at}\right)+\frac{u}{a}\left[x_{\left(0\right)}^{\left(1\right)}⊃x_{\left(1\right)}^{\left(0\right)}\right]$$

(6) $${\hat{x}}^{\left(0\right)}\left(t\right)={\hat{x}}^{\left(1\right)}\left(t\right)-{\hat{x}}^{\left(1\right)}\left(t-1\right)$$

where *a* represents the development gray number, *u* represents the endogenous control gray number, and *t* is the prediction time.

### 2.4. Data Sources

An analysis of China’s *EF* including data on Yangtze River urban agglomeration from 2013 to 2017 comes from the *Statistical Yearbook*, *Agricultural Yearbook*, and *Energy Yearbook*, which was published by eight cities’ statistical bureaus. The missing data were mainly estimated by an interpolation of the adjacent year. Among them, the two key parameters equivalence factors and yield factors in the *EF* method are determined according to the study area. Based on the output of various types of consumer goods in the *EF* account for indicators provided by the national data network in the National Data Network 2010 and the calorific value data of each product in the *Agricultural Economics Manual (Revised)*, the equivalence factors of various types of land used in the country are calculated (Table 1). For yield factor, it is obtained through a comparison between *Jiangsu Statistical Yearbook 2011* by counties with the national production data.

## 3. Results and Analysis

### 3.1. Evaluation of Ecological Footprint and Ecological Carrying Capacity for Yangtze River Urban Agglomeration from 2013 to 2017

#### 3.1.1. Calculation of the Ecological Footprint of Yangtze River Urban Agglomeration in 2017

According to the statistical yearbook released by the eight cities’ statistical bureaus of Yangtze River urban agglomeration, the calculation method of the *EF* in Section 2 is used to calculate and analyze the *EF* of the eight cities in 2017. The calculation is divided into two parts: biological resource consumption and energy consumption.

(1) Consumption of biological resources

We divide the consumption of biological resources into crop products, animal products, forest products, and other projects, and use the world average production data on biological resources calculated by the United Nations (UN) Food and Agriculture Organization in 1993 to convert the production area of biological resources. The calculation results of the *EF* consumption of the biological resources of eight cities in 2017 are shown in Table 2.

(2) Consumption of energy resources

The energy consumption of Yangtze River urban agglomeration mainly includes washing coal, other coal washing, raw coal, coke, gasoline, kerosene, diesel, other fuels, fuel oil, other petroleum products, liquefied petroleum gas, natural gas, other gas, electricity, and heat, and a total of 15 species. For primary energy consumption such as raw coal and natural gas, it is unified into fossil fuel land based on the global average calorific value and conversion factor. For secondary energy power and heat, the *EF* is transformed into a fossil fuel land that absorbs CO_2_ from coal-fired power generation as an indirect coal consumption *EF*. For the built-up area, the comprehensive calculation of each year’s urban built-up area includes all completed and uncompleted land. Thus, the *EF* of various energy resources consumption in eight cities in 2017 are shown in Table 3.

#### 3.1.2. Calculation of Ecological Carrying Capacity for Yangtze River Urban Agglomeration in 2017

According to the biological production area per capita that Yangtze River urban agglomeration can actually provide in 2017, the *EF* per capita and *EC* per capita are calculated and compared. See Table 4 and Table 5 for details.

The equivalence area per capita is calculated by Area per capita (hm^2^/person) * Equivalence factor. The *EC* per capita refers to *EC*/Total resident population.

From Table 4 and Table 5, the *EF* per capita for Yangtze River urban agglomeration in 2017 is 1.2611 hm^2^, while the *EC* per capita is only 0.3595 hm^2^; after deducting 12% of that reserved for biodiversity conservation (0.0431 hm^2^), the available *EC* per capita is 0.3164 hm^2^, and the ED per capita reaches 0.9447 hm^2^. The *EF* of the region is nearly four times greater than its *EC*, indicating that the supply of land resources in the region is far from meeting the demand, and the ecological environment is in an unsustainable state.

Among them, the deficit in the water area is the most serious, followed by pasture and arable land. This is closely related to the uncoordinated development of the Yangtze River urban agglomeration. Taking Subei as an example, the northern part of Jiangsu is a relatively backward economic development area. The level of the primary industry is relatively high, accounting for 11.61% [39]; the proportion of planting industry is too large; it is basically a farming society, which leads to excessive *EF* in the water area, pasture, and arable land. At the same time, the security risks of the industrial structure system that is dominated by the petrochemical industry are prominent, which will lead to increased pollution of the water, land, and other resources in the region, and further reduce the regional *EC*, thus affecting regional sustainable development. Only the forest and built-up area are in a surplus, but they also tend to be saturated, which should also be taken seriously.

#### 3.1.3. The Dynamic Trend of the Ecological Footprint for Yangtze River Urban Agglomeration from 2013 to 2017

**Figure 2:**
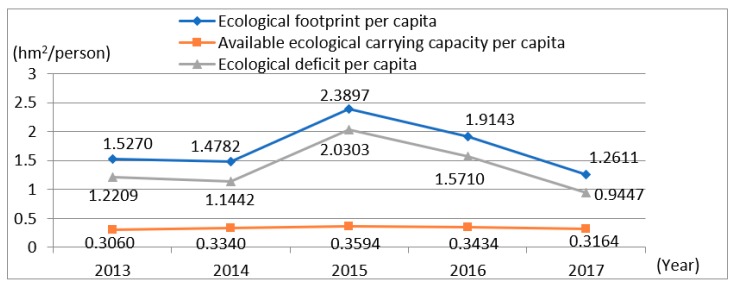
The broken line graph of the ecological footprint and ecological carrying capacity for Yangtze River urban agglomeration from 2013 to 2017.

Using the abovementioned *EF* calculation method, based on the statistical yearbook data of the eight cities (2013–2017), we calculate the *EF*, the *EC*, and the ED for Yangtze River urban agglomeration from 2013 to 2017. Based on this, the change in trend of the overall *EF* from 2013 to 2017 was obtained (Table 6), and the broken line graph of the demand and supply of the *EF* from 2013 to 2017 was obtained (Figure 2).

The *EF* demand per capita is calculated by Total *EF* per capita of forest + Pasture + Water area + Built-up area + Arable land + Fossil-energy land Equivalence factor. The available *EC* per capita refers to *EC* * 0.88 (because 12% of the biodiversity conservation land should be reserved after deduction).

From Table 6, it can be seen that the *EF* per capita for Yangtze River urban agglomeration increased from 1.5270 hm^2^ in 2013 to 2.3897 hm^2^ in 2015 and decreased to 1.2611 hm^2^ in 2017. In the same period, the available *EC* per capita increased from 0.3060 hm^2^ in 2013 to 0.3594 hm^2^ in 2015 and then decreased to 0.3164 hm^2^ in 2017. During the study period, the *EF* per capita and the available *EC* per capita for Yangtze River urban agglomeration basically developed in the same direction, resulting in the ED per capita increasing from 1.2209 hm^2^ in 2013 to a peak of 2.0303 hm^2^ in 2015 and falling to 0.9447 hm^2^ in 2017.

From Figure 2, the change in *EC* from 2013 to 2017 is generally slight, and the ecological deficit is caused mainly by the huge change of the *EF*. At the same time, we can see that since 2015, the ED has also been significantly reduced under the almost constant *EC*. The reason for this trend is that the Jiangsu Provincial Government released the “Implementation Opinions on Accelerating the Construction of Ecological Civilization” in 2015 and vigorously advanced the “Seven Actions” of the ecological civilization construction project. During this period, Jiangsu province strengthened the control of the ecological space and allocated 1.5 billion yuan for provincial ecological compensation, which was used to comprehensively rectify 51,800 urban environmental projects and 90.4% of sewage treatment plants and achieved comprehensive facility coverage for urban and rural waste transportation systems [40]. Therefore, during 2016, the *EF* for Yangtze River urban agglomeration has dropped significantly, and the environmental quality has maintained a good momentum of overall improvement. It can be seen that the government’s strengthening of environmental regulations will effectively reduce the *EF* of the region and further decrease the ED.

### 3.2. Prediction of the Ecological Footprint and the Ecological Carrying Capacity for Yangtze River Urban Agglomeration from 2018 to 2022

#### Predictions

**Figure 3:**
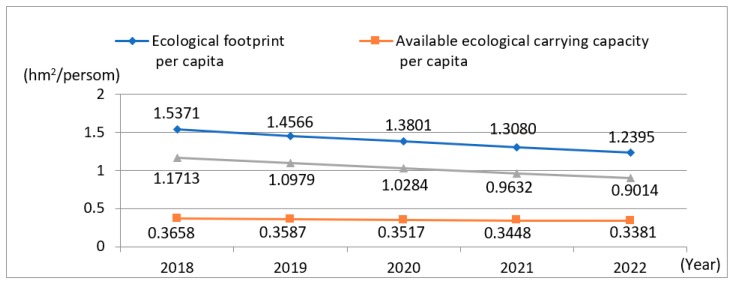
Trends in the supply and demand of ecological footprints from 2018 to 2022.

Based on the *EF* per capita and available *EC* per capita for Yangtze River urban agglomeration from 2013 to 2017, the GM (1,1) model was utilized to predict the ED in the study area from 2018 to 2022. The prediction model is shown in Table 7. The trend of the changes in the supply and demand of the *EF* from 2018 to 2022 is shown in Figure 3.

The model mainly tests the prediction accuracy by calculating the relative error. When the absolute value of the relative error is less than 3%, the accuracy is very high and the relative error is smaller. It can be seen from Table 7 that the relative error of the *EF* per capita is 1.51%, and the relative error of the available *EC* per capita is 3.1%. This shows that under the condition of sufficient data, the prediction results can be more accurate using the Grey model.

From Figure 3, in 2018, the *EF* per capita and the available *EC* per capita for Yangtze River urban agglomeration will be expected to be 1.5371 hm^2^ and 0.3658 hm^2^, respectively, and the ED per capita will be 1.1713 hm^2^, which will be another deficit peak after the ED per capita of 2.0303 hm^2^ in 2015. The reason for this phenomenon is that the acceleration of the urbanization process for Yangtze River urban agglomeration will lead to an increase in the demand for resources. At the same time, the influx of population will lead to a large increase in urban residents, increasing the *EF* of human beings. In the future, it will be necessary to control the urban population and reasonably guide the consumption of residents to alleviate the ecological pressure.

However, in the later period until 2022, as seen from Figure 3, the ED will gradually decline. From the overall downward trend, the reduction of the *EC* brought about by the urbanization process will merit more and more attention. The construction of ecological governance proposed by the Chinese government will provide hard targets for the subsequent ecological construction of Yangtze River urban agglomeration, forcing enterprises to cut energy consumption and decrease pollution, thereby effectively reducing the ED and easing the contradiction between man and nature.

## 4. Conclusions

### 4.1. Main Conclusions

The research uses the *EF* model to evaluate the *EF* per capita, the *EC* per capita, and the ED per capita for Yangtze River urban agglomeration during 2013–2017. The evaluation results show that the *EF* per capita has increased from 1.5270 hm^2^ in 2013 to 2.3897 hm^2^ in 2015, and dropped to 1.2611 hm^2^ in 2017. In the same period, the available *EC* per capita continued to increase from 0.3060 hm^2^ in 2013 to 0.3594 hm^2^ in 2015, reaching its peak, and then decreased to 0.3164 hm^2^ in 2017. During the study period, the *EF* per capita and the available *EC* per capita for Yangtze River urban agglomeration developed in the same direction, causing the ED per capita to increase from 1.2209 hm^2^ in 2013 to a peak of 2.0303 hm^2^ in 2015, and then fall to 0.9447 hm^2^ in 2017, and the *EF* has been in an ED, which is an unsustainable development. This pattern reflects the contradiction between the ecological supply and the ecological demand of the Yangtze River urban agglomeration, and it is necessary to input the *EF* from the outside to ease local contradictions. At the same time, we find that the changes in the *EC* are not substantial, and the ED is mainly caused by huge changes in the *EF*.

Then, based on the *EF* per capita and the available *EC* per capita for Yangtze River urban agglomeration from 2013 to 2017, the GM (1,1) model was used to predict the ED in the study area from 2018 to 2022. The prediction results show that in 2018, the ED per capita for Yangtze River urban agglomeration will be 1.1713 hm^2^, which will be another peak after the ED per capita of 2.0303 hm^2^ in 2015. However, in the later stage until 2022, the ED per capita will decline year by year. From another perspective, the ED has always existed, and the prospect of improvement is not optimistic. The relevant governments should attach great importance to it and resolutely implement ecological environmental protection systems to reduce the *EF*, increase resource utilization, and boost regional sustainable development.

### 4.2. Suggestions

The research findings suggest that in order to promote the sustainable development of the Yangtze River urban agglomeration, we should not blindly increase the *EC* from the aspects of increasing environmental protection investment and so on. Instead, we should start with reducing the *EF*, and then increase the *EC*. This provides effective macro guidance for the coordinated development of economic development and environmental protection in the Yangtze River urban agglomeration and captures the main contradictions affecting the sustainable development of the Yangtze River urban agglomeration. Therefore, we further propose the following specific measures:

(1) It is necessary to control the scale of the population appropriately and increase environmental protection publicity. The increase in population is one of the important factors for the increase of the *EF*. Therefore, the study area needs to control the population. At the same time, we should raise residents’ awareness of environmental protection, advance environmental education and publicity work for residents in rural areas, and encourage green lifestyles and consumption.
(2) Investment in science and technology should increase and energy efficiency should be enhanced. By increasing investment in science and technology, encouraging new and renewable energy sources, and enhancing energy efficiency, we will gradually reduce the proportion of fossil fuels, such as raw coal, in energy consumption and optimize the energy structure.
(3) Paving a new road to industrialization. It should learn advanced technologies and constantly adjust the industrial strategic layout. At the same time, the traditional industries in Taizhou, Yangzhou, and Nantong should also be transformed to cultivate new economic growth points and foster product groups with local characteristics. On the other hand, we should stimulate the transfer of industries to high-end value chains and encourage the development of service industries.
(4) Protecting water, pasture, and arable land. Yangtze River urban agglomeration has a high demand for water, pasture, and arable land. Therefore, it is necessary to speed up the construction of the water conservancy infrastructure and strengthen the flood control and drought prevention capacity in this area. In particular, we should protect the ecological system of Taihu Lake, the largest lake in the province, and enhance its ability to conserve water and soil, as well as lower surface pollution, especially by reducing chemical pollution emissions from industrial enterprises in the Taihu Basin. At the same time, it is also necessary to return farmland to the lake and forests to improve vegetation coverage and strengthen the protection of agricultural land, especially the rehabilitation of soil and the restoration of degraded land. Finally, we should optimize the delineation of three control lines for ecological protection: the red line for ecological protection, permanent basic farmland, and the border for urban development; strictly protect existing farmland; and advance the comprehensive treatment of desertification, stony desertification, and soil erosion.
(5) Strengthening environmental monitoring and enforcement. First, the optimization and adjustment of the provincial ecological red line area should be initiated, and the ecological red line protection plan, management, and control measures and compensation policies within the scope of demarcation should be formulated. At the same time, environmental law enforcement should be strengthened, and the supervision and management of the provincial ecological red line should be assessed regularly. Strengthening coordination and communication among environmental departments, governments at higher levels, and local county and municipal departments should work together to crack down on various environmental violations in accordance with the law and gradually clean up some of the polluting enterprises. Finally, the environmental supervision departments at all levels of the Yangtze River urban agglomeration should strengthen the fostering of environmental supervision professionals in key industries, such as the chemical and medicine industry, to improve the efficiency of environmental inspection.

## Figures and Tables

**Table 1:** Description of land types in the ecological footprint account.

Land Type	Main Application	Equivalence Factor
Arable land	Provide crops	2.51
Forest	Provide forest products	1.26
Pasture	Provide livestock products	0.46
Water area	Provide aquatic products	0.37
Fossil-energy area	Absorb carbon dioxide released by humans	1.26
Built-up area	Land for human life and construction	2.51

Note: (1) The global average bio-capacity is 1. (2) Twelve percent of the deductions are for biodiversity conservation land. (3) In real life, people do not set aside land for absorbing carbon dioxide.

**Table 2:** Calculation of the ecological footprint of the biological resources for Yangtze River urban agglomeration in 2017.

	Nanjing (t)	Nantong (t)	Zhenjiang (t)	Yangzhou (t)	Wuxi (t)	Suzhou (t)	Taizhou (t)	Changzhou (t)	Global Average Production (kg·hm·10^−2^)	Total Ecological Footprint (hm^2^/person)	Ecological Footprint Per capita (hm^2^)	Type of Cultivated Land
Paddy	768,375	1,605,397	776,916	287,290	271,938	665,632	371,938	864,312	2744	2,045,116	0.0472	Arable land
Wheat	234,638	961,731	347,308	1,033,943	197,818	285,282	197,818	389,792	2744	1,329,566	0.0307	Arable land
Corn	40,952	330,116	33,594	12,337	16,994	9294	12,495	27,895	2744	176,267	0.0041	Arable land
Beans	14,554	129,871	16,105	53,770	7032	4544	8819	15,308	1856	134,700	0.0031	Arable land
Potato	21,400	19,596	12,292	14,636	14,957	13,885	7032	1024	12,607	8315	0.0002	Arable land
Cotton	3071	23,797	910	1144	1365	500	520	371	1000	31,678	0.0007	Arable land
Oil	74,337	358,183	58,291	68,669	2212	13,409	2212	24,144	1856	324,061	0.0075	Arable land
Vegetables	2,149,606	4,390,975	1,766,902	2,068,921	45,896	39,896	45,896	2,714,920	18,000	734,612	0.0169	Arable land
Melon and fruit	243,237	577,994	144,101	92,825	176,222	95,595	176,222	103,312	18,000	89,417	0.0021	Arable land
Pig	53,150	256,366	47,686	98,677	51,665	61,778	51,665	39,842	74	8,930,122	0.2060	Pasture
Cow	832	313	12,716	638	38	467	38	679	33	476,394	0.0110	Pasture
Sheep	2603	26,518	19,075	1866	319	1982	319	39,842	33	2,803,758	0.0647	Pasture
Aquatic products	223,098	890,285	99,896	401,183	13,995	254,918	197,393	166,351	29	77,486,862	1.7877	Waters
Milk	74,721	21,441	18,046	12,665	26,290	102,957	26,290	19,823	502	602,058	0.0139	Pasture
Honey	259	296	365	652	203	397	203	459	50	56,680	0.0013	Pasture
Egg	65,367	447,700	27,775	137,404	27,017	38,030	27,017	39,624	400	2,024,835	0.0467	Pasture
Tea	1540	0	1756	6861	6507	361	0	2585	566	23,737	0.0005	Forest

**Table 3:** Calculation of the energy ecological footprint for Yangtze River urban agglomeration in 2017.

	Nanjing (GJ)	Wuxi (GJ)	Suzhou (GJ)	Changzhou (GJ)	Zhenjiang (GJ)	Nantong (GJ)	Yangzhou (GJ)	Taizhou (GJ)	Global Average Energy Footprint (GJ·hm^−2^)	Convert Coefficient (GJ·t^−1^)	Total Consumption (t)	Consumption Per Capita (GJ/person)	Ecological Footprint Per Capita (hm^2^/person)	Ecological Productive Land Type
Raw coal	27,859,054	25,585,565	52,930,655	10,259,787	18,853,868	21,035,064	10,472,274	15,794,328	55	20.9340	182,790,595	4.2172	0.0767	Fossil fuel land
Washed coal	5,853,334	2,278,006	7,678,792	0	589,212	0	0	92,656	55	26.3440	16,492,000	0.3805	0.0069	Fossil fuel land
Other coal washing	1351	0	5739	0	16	0	127,900	0	55	8.3630	135,006	0.0031	0.0001	Fossil fuel land
Coke	6,556,289	4,048,528	12,288,290	5,097,035	602,498	145,694	0	35,106.89	55	28.4700	28,773,441	0.6638	0.0120	Fossil fuel land
Other fuel	0	654	11,260	0	0	0	973,226	0	55	8.3630	985,140	0.0227	0.0004	Fossil fuel land
Gasoline	27,186	26,571	66,250	9251	17,312	36,007	32,259	14,989.41	93	43.1240	229,825	0.0053	0.0001	Fossil fuel land
Kerosene	248	840	2442	105	44,234	4085	299	6098.19	93	43.1240	58,351	0.0013	0.0001	Fossil fuel land
Diesel	72,969	63,961	151,720	12,528	14,349	51,910	56,542	51,012.43	93	42.7050	474,991	0.011	0.0001	Fossil fuel land
Fuel oil	6527	105,213	123,981	0	2084	28,937	5333	146,327.89	71	50.1600	418,403	0.0097	0.0001	Fossil fuel land
Other petroleum products	12,128,828	2469	3753	0	1767	0	47,573	683.63	71	50.1600	12,185,074	0.2811	0.0040	Fossil fuel land
Liquefied petroleum gas	366,100	2273	8820	1128	22,337	71,849	3290	28,020.12	71	50.1600	503,817	0.0116	0.0002	Fossil fuel land
Natural gas	236,540	224,033	401,984	143,123	0	16,869	81,930	0	93	38.9790	1,104,479	0.0255	0.0003	Fossil fuel land
Electricity	3,126,235	4,092,900	9,196,802	4,299,322	1,583,368	1,353,245	1,563,203	1,761,311.9	1000	11.8400	26,976,387	0.6224	0.0006	Built-up area
Heat	95,201,693	77,784,760	108,628,412	27,632,015	25,559,810	54,357,115	16,960,399	201,282,559	1000	29.3400	607,406,763	14.0135	0.0140	Built-up area

**Table 4:** The demand of ecological footprint for Yangtze River urban agglomeration in 2017.

Land Type	Area per Capita (hm^2^/person)	Equivalence Factor	Equivalence Area per Capita (hm^2^/person)
Arable land	0.1104	2.51	0.2771
Pasture	0.3436	0.46	0.1581
Forest	0.0005	1.26	0.0007
Built-up area	0.0146	2.51	0.0367
Fossil energy land	0.1009	1.26	0.1271
Water area	1.7877	0.37	0.6615
Total			1.2611

**Table 5:** The Supply of ecological footprints for Yangtze River urban agglomeration in 2017.

Land Type	Area (hm^2^)	Equivalence Factor	Yield Factor	Ecological Carrying Capacity (hm^2^)	Ecological Carrying Capacity per Capita (hm^2^/person)
Arable land	2,099,120	2.51	1.66	8,746,193	0.2018
Pasture	3,662,319	0.46	0.19	320,086.7	0.0074
Forest	224,336	1.26	0.91	257,223.7	0.0059
Built-up area	1,431,049	2.51	1.66	5,962,609	0.1376
CO_2_ absorption land	0	1.26	0	0	0
Water area	806,220	0.37	1	298,301.4	0.0069
Total	8,223,044			15,584,413.8	0.3595

**Table 6:** Trends in ecological footprint per capita, ecological carrying capacity per capita, and ecological deficit per capita for Yangtze River urban agglomeration from 2013 to 2017.

Year	Ecological Footprint per Capita (hm^2^/person)	Ecological Carrying Capacity per Capita (hm^2^/person)	Available Ecological Carrying Capacity per Capita (hm^2^/person)	Ecological Deficit per Capita (hm^2^/person)
2013	1.5270	0.3478	0.3060	1.2209
2014	1.4782	0.3795	0.3340	1.1442
2015	2.3897	0.4084	0.3594	2.0303
2016	1.9143	0.3902	0.3434	1.5710
2017	1.2611	0.3595	0.3164	0.9447

**Table 7:** Prediction model of the ecological footprint per capita and the available ecological carrying capacity per capita for Yangtze River urban agglomeration.

Forecasting Object	Grey Forecasting Model	Model Checking	Relative Error
Ecological footprint per capita	$\hat{x}\left(t+1\right)$ = 37.922 − 36.395 × exp(−0.0537948 × *t*)	excellent	1.51%
Available ecological carrying capacity per capita	$\hat{x}\left(t+1\right)=$ 20.6091 − 20.2613 × exp(−0.0197318 × *t*)	good	3.10%

Note: *e* is a constant value of 2.71828, *t* represents the predicted time.
